# Smartphone image dataset for turmeric plant leaf disease from Bangladesh spice fields

**DOI:** 10.1016/j.dib.2025.112184

**Published:** 2025-10-16

**Authors:** Jubaer Ahmed, Md. Riyad Hossain, Raiyan Gani, Mohammad Rifat Ahmmad Rashid, Md. Mahamudur Rahman, Tasfia Binte Jahangir, Md. Samir Hossain

**Affiliations:** Department of Computer Science and Engineering, East West University, Aftabnagar, Dhaka, Bangladesh

**Keywords:** Disease identification, Dataset collection, Turmeric leaf diseases, Agricultural Challenges

## Abstract

Agriculture is key to sustaining life and economic development, and crops like turmeric are essential for everyday application and economic viability. Turmeric crops are very prone to foliar disease, which has a great impact on yield and quality. Early detection of the diseases is of great significance to farming practitioners since manual observation is generally time-consuming and unreliable. To surpass this challenge, a comprehensive dataset has been developed to facilitate the generation of an automatic disease recognition system. The dataset comprises 865 images of original turmeric leaves and 3496 images of augmented turmeric leaves, both infected and healthy, with four classes of diseases: aphid attack, blotch, leaf spot, and healthy leaves. All the leaves were captured from different angles to offer variability and clarity, with particular emphasis on high-quality and diversified data. Through this dataset, a precise and efficient identification process can be realized, which will aid agriculture practitioners in recognizing diseases at an early stage and reducing crop losses. This paper seeks to improve agricultural productivity, crop quality, and the overall growth and sustainability of the agricultural economy using state-of-the-art deep learning models, such as EfficientNetB7 and ResNet152, for precise and interpretable disease classification. The proposed approach achieves high accuracy, with EfficientNetB7 attaining 98.67 % and ResNet152 reaching 97.87 %. Additionally, this research lays the groundwork for scalable and affordable disease detection technology, allowing agricultural practitioners to maximize crop yield and achieve long-term food security using smart tools.

Specifications Table

**Table:** 

Subject	Plant Pathology is a branch of Agriculture
Specific subject area	Turmeric Plant Leaf Disease Identification and Classification.
Type of data	Raw and processed leaf images are available in JPG format, with resolutions of 4000×3000 and 3000×4000 (original dataset) pixels and 224×224 (augmented dataset) pixels.
Data collection	Samples of turmeric leaf disease were collected through a comprehensive field study, observations at four locations in the Pabna district, encompassing four disease categories: aphid infestation, blotch, leaf spot, and healthy leaves. The diseased and healthy leaves were differentiated through expert consultation and reference to authoritative online sources. Leaf samples were photographed from five angles against a white background to minimize extraneous visual elements and optimize image quality. A Redmi K20 smartphone with high-resolution capabilities was employed to ensure superior image quality. The resulting collection of 865 images proved to be a valuable dataset for training deep learning models.
Data source location	1. Shibpur village turmeric field in Pabna (latitude: 24.049915082362332, longitude: 89.36065124780326) 2. Kodimbokdi village turmeric field in Pabna (latitude: 24.059176119933124, longitude: 89.34977080928246) 3. Ekdonto village turmeric field in Pabna (latitude: 24.071123635779465, longitude: 89.34558471048882) 4. Tebunia village turmeric field in Pabna (latitude: 24.070634252228754, longitude: 89.20332505175169)
Data accessibility	Repository name: Mendeley Data Data identification number: DOI: 10.17632/jtttfbx342.1 Direct URL to data: https://data.mendeley.com/datasets/jtttfbx342/1

## 1 Value of the Data

• This dataset generates a wealth of visual information on leaf diseases of turmeric, which is a good resource to train machine learning models. The models can be constructed to differentiate well between healthy and diseased leaves so that the diseases can be diagnosed early and accurately in agricultural applications.
• Using machine learning models trained on this data, farmers and agricultural specialists can forecast possible outbreaks of disease. Such predictive power can enable early intervention, which could minimize crop losses and encourage sustainable agriculture.
• The database serves as a reference for advanced research, providing researchers and agronomists with fundamental details regarding disease formation, environmental conditions, and therapeutic results. The findings have the potential to advance new agriculture practices, with an application of possibly integrating increased disease resistance into crops.
• Early and accurate disease diagnosis, facilitated by machine learning algorithms trained on this dataset, could result in healthier turmeric crops and improved harvests. This may have financial impacts on farmers' livelihoods as well as improving food security in turmeric-dependent nations.
• The models and methods found here can be scaled to be used in other regions of the Earth and in other varieties of crops. This scalability enhances the transferability of turmeric disease studies and can lead to worldwide innovation in the identification of and prevention against plant diseases.

## 2 Background

The turmeric leaf disease dataset is crucial to further developing precision agriculture and plant disease diagnosis. Well-annotated image data allows robust machine learning models for early classification and detection of foliar diseases. This allows timely action, avoiding loss of yield and chemicals. Such datasets are particularly vital for such crops as turmeric, which are subjected to several biotic stresses across a range of environmental conditions. On the other hand, turmeric is an economically and medically valuable spice crop that fetches a high price. However, it is often infected with leaf diseases that can drastically influence yield and quality. The traditional practices by farmers include hand scouting of affected plants, which is time-consuming and prone to error. The lack of proper diagnostic methods often leads to disease misdiagnosis, poor disease control, and unnecessary economic losses. With advancements in machine learning (ML) and computer vision, automated detection and classification of diseases have become increasingly feasible [1]. The technologies provide high-accuracy, high-volume, and real-time solutions through the analysis of high-resolution images of plant leaves. Current deep learning methods, such as Convolutional Neural Networks (CNNs), transfer learning, and image processing, have proved to have superior success in accurate disease classification. CNNs revolutionized plant disease detection based on their ability to learn elaborate spatial patterns from images. Transfer learning also enhances these models further by using pre-trained networks over vast amounts of data so that the models are fine-tuned to specific plant diseases using relatively small datasets. Image augmentation techniques are also employed to augment the diversity of training data synthetically to increase model strength and avoid overfitting. Such deep learning algorithms have been proven to carry out plant disease diagnosis with high accuracy, for instance, as demonstrated in [2], where a smartphone image dataset was used to distinguish healthy and diseased papaya leaves. EfficientNetB7 and ResNet-152 have also shown remarkable performance in plant disease detection [3,4]. EfficientNetB7, with its compound scaling approach, balances network depth, width, and resolution, making it highly efficient while maintaining high accuracy. ResNet-152, with its deep residual learning framework, is effective in handling complex plant disease patterns by mitigating vanishing gradient issues and enhancing feature extraction. These models have been widely adopted in agricultural applications for their robustness and accuracy in disease classification. Some research has also explored ML-based plant disease detection with domain-specific datasets. For instance, in [5], a 2137 original and 7000 augmented image dataset was developed for the detection of cotton leaf disease and achieved 96.03 % accuracy using Inception V3. Similarly, research in [6] was concerned with classifying bean and cowpea leaf disease with a dataset consisting of 4467 images from 8 disease classes for enhancing plant disease detection through ML models. These advances in model performance and dataset creation have paved the way for the development of more precise and reliable systems for classifying plant diseases. Building upon these advances, this work proposes an extensive turmeric leaf disease dataset, with a variety of disease classes, including leaf spot, blotch, and pest damage. Employing deep learning models, it is suggested to the development of an accurate and robust classification system for turmeric leaf diseases. Using real-world, high-resolution images, our dataset provides accurate model training and disease identification. The results achieved through this research will assist in ensuring sustainable turmeric farming, enabling early intervention, low transmission of diseases, and improved crop yield. Further, the process can be replicated for other spice crops, thus expanding global reach through ML-based agricultural solutions.

## 3 Data Description

**Fig. 1:**
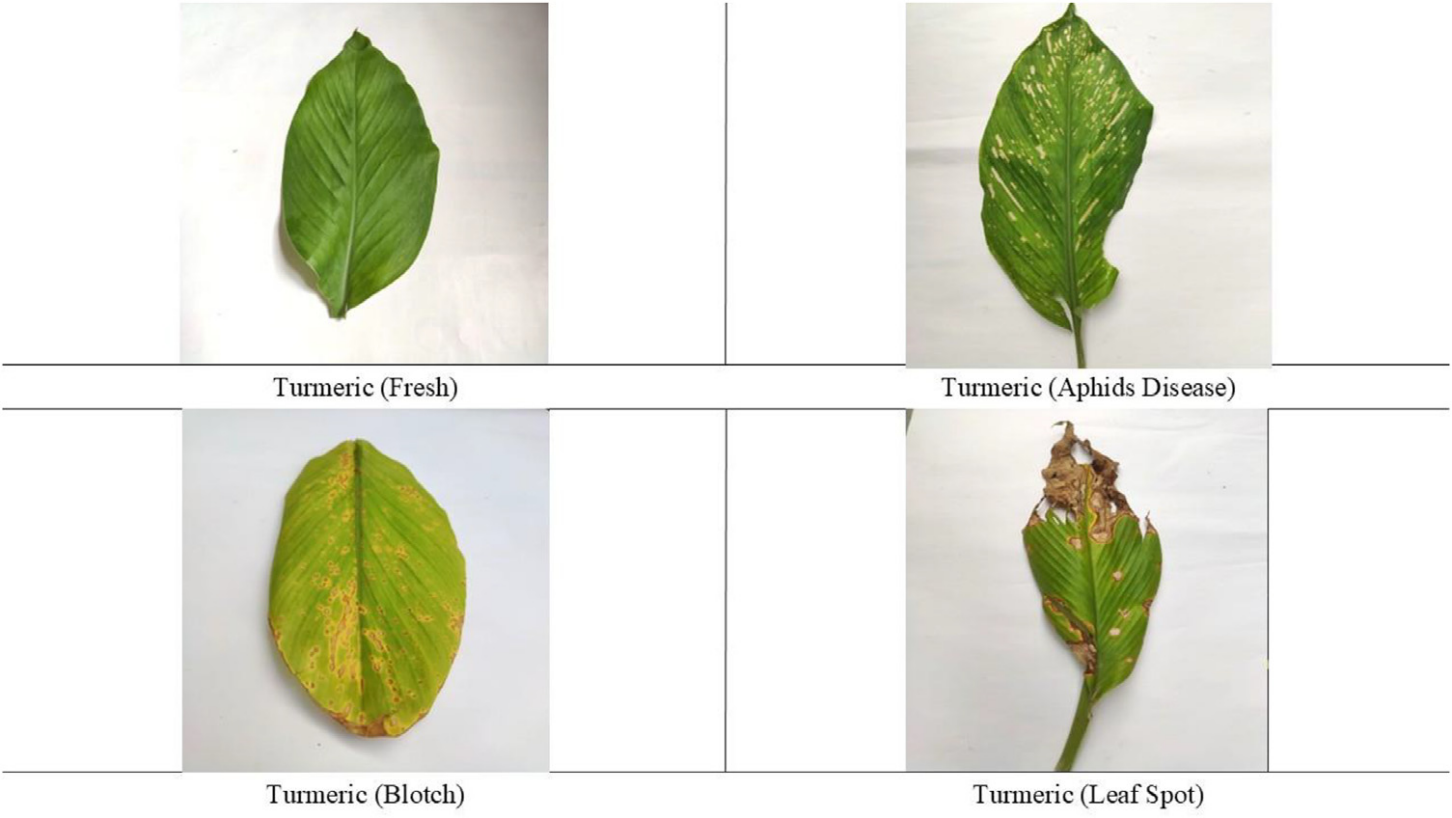
Sample images of turmeric leaf from every class.

The dataset [7] comprises a meticulously curated collection of 865 original high-resolution images of turmeric leaves, systematically gathered from agricultural regions in Pabna, Bangladesh, and also provides 3496 augmented images. All the images are saved in JPG format. The original image dataset contains 4000×3000 pixels, 3000×4000 pixels images, and the augmented dataset contains 224×224 pixel images. These images have been carefully captured and formatted to support research in machine learning and deep learning applications. The dataset is structured in a standardized format, ensuring comprehensive representation of both diseased and healthy turmeric leaves. To enhance usability, images are systematically categorized into four distinct classes: aphid disease, blotch, leaf spot, and healthy leaf, as illustrated in Fig. 1. This structured organization facilitates efficient navigation and accessibility for researchers. By providing a diverse set of labeled images, this dataset serves as a valuable resource for precision agriculture, enabling the development of advanced computational models for automated plant disease detection and diagnosis. Future enhancements may include additional disease categories and expanded image collections to further strengthen its applicability in agricultural research further.

**Fig. 2:**
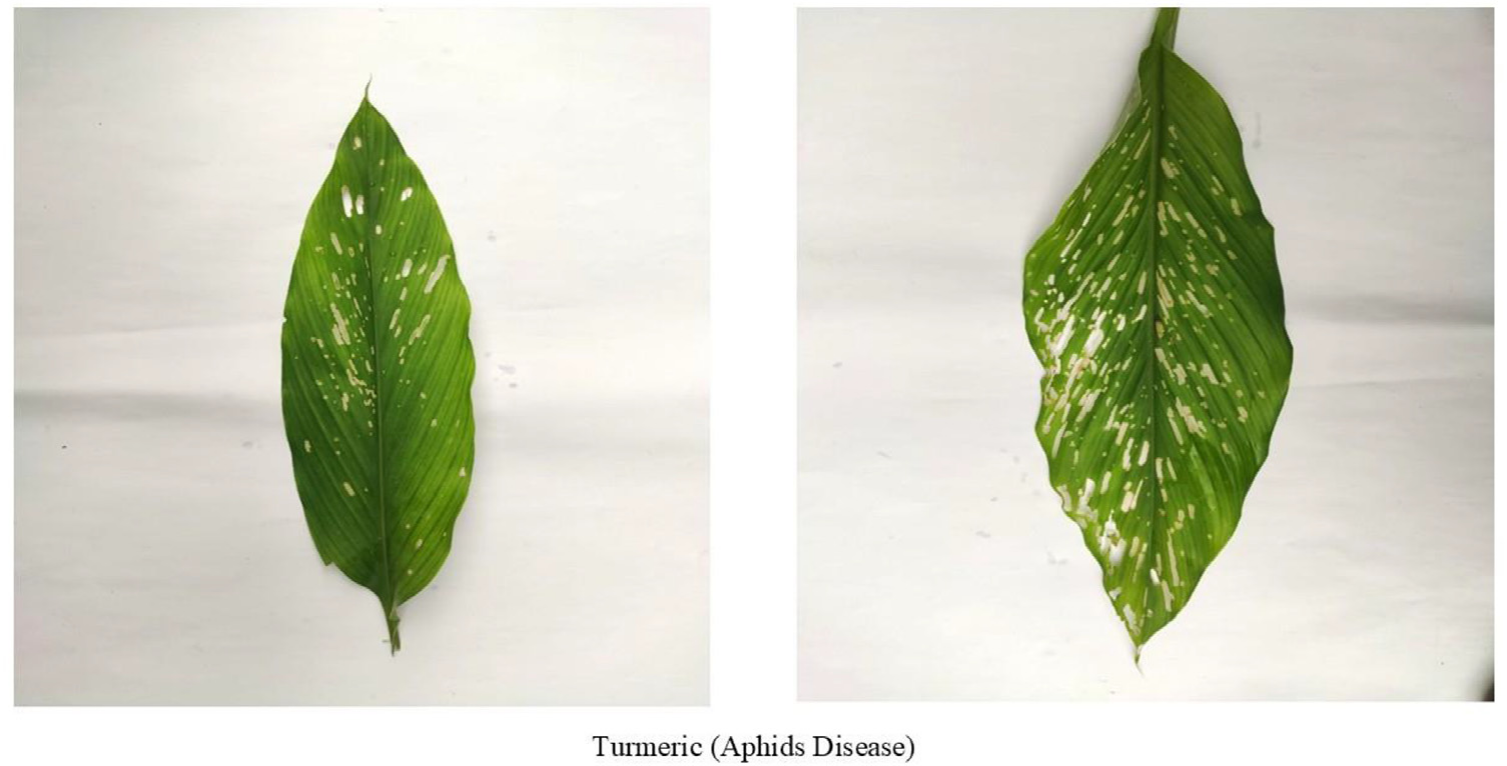
Turmeric leaf affected by Aphid disease.

Fig. 2 depicts turmeric aphid disease, small Aphididae family insects, which destroy turmeric leaves by injecting plant tissues with pointed mouthparts and ingesting plant sap, resulting in curled leaves, folded or wrinkled leaves, chlorosis, necrosis, and disease-causing virus transference. The honeydew droppings facilitate fungal growth and suppress photosynthesis, weaken plant vigor, and induce early leaf senescence [8]. Aphids are efficient disease transmitters due to specialized apparatuses in the mouth [9] and have high population rates under the regulation of temperature or humidity. They are host plant specific or infect variable crops. Natural enemies, ladybugs, and lacewings control the population of aphids, reducing chemical pesticide use [10].

**Fig. 3:**
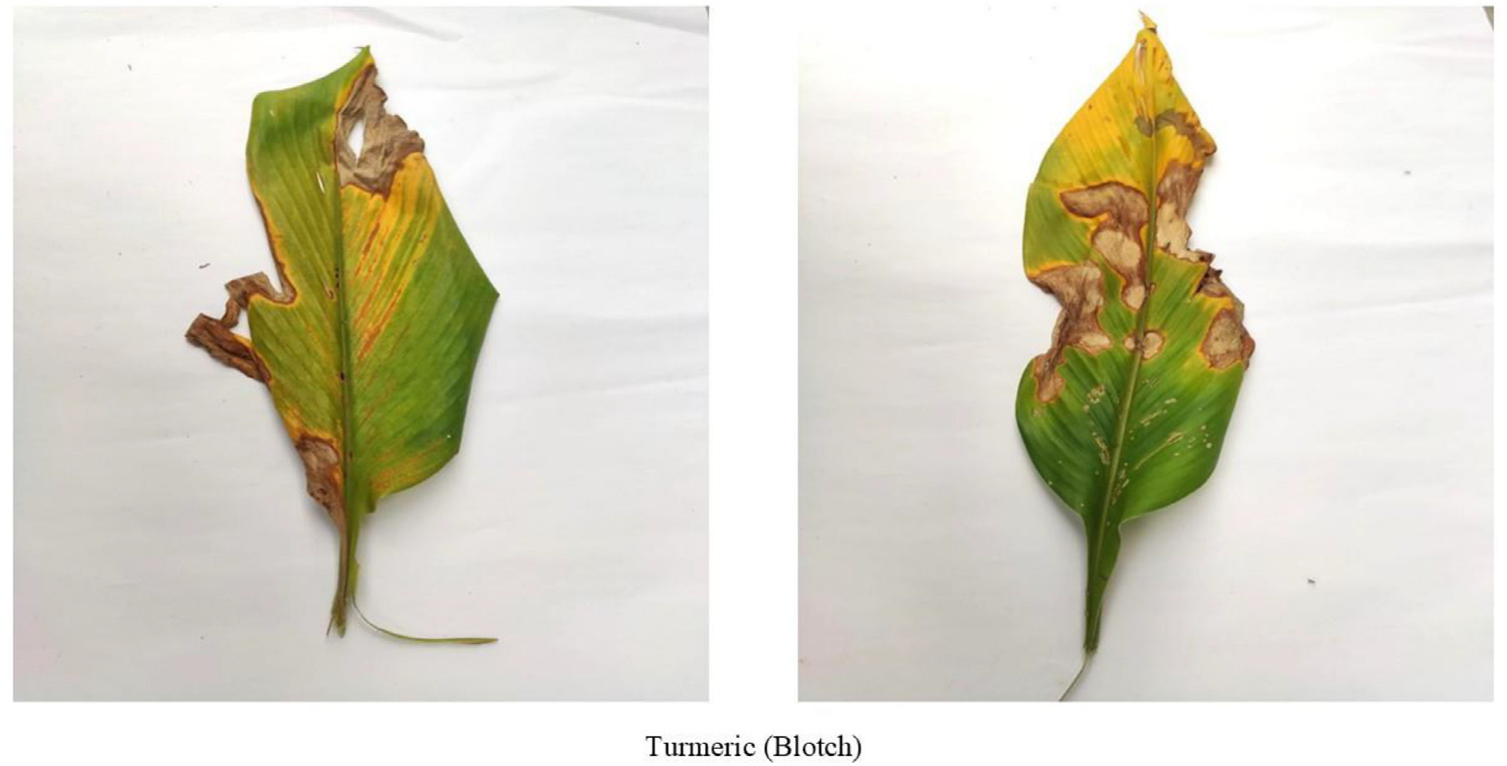
Turmeric leaf affected by Blotch disease.

Fig. 3 illustrates turmeric blotch, Taphrina maculans-induced leaf blotch, spreading quickly through wind-born spores through wind, water, and insects under moderate humid conditions [11]. It first occurs as minute brown spots which grow into darker spots [12], affecting the structure of the leaves, preventing the functioning of chlorophyll, and photosynthesis. This causes stunted growth, early-leaf senescence, and yield reduction in turmeric crops. Successful control measures comprise crop replanting, field sanitation, planting under optimum conditions, the use of fungicides, and the use of resistant or biocontrol measures.

**Fig. 4:**
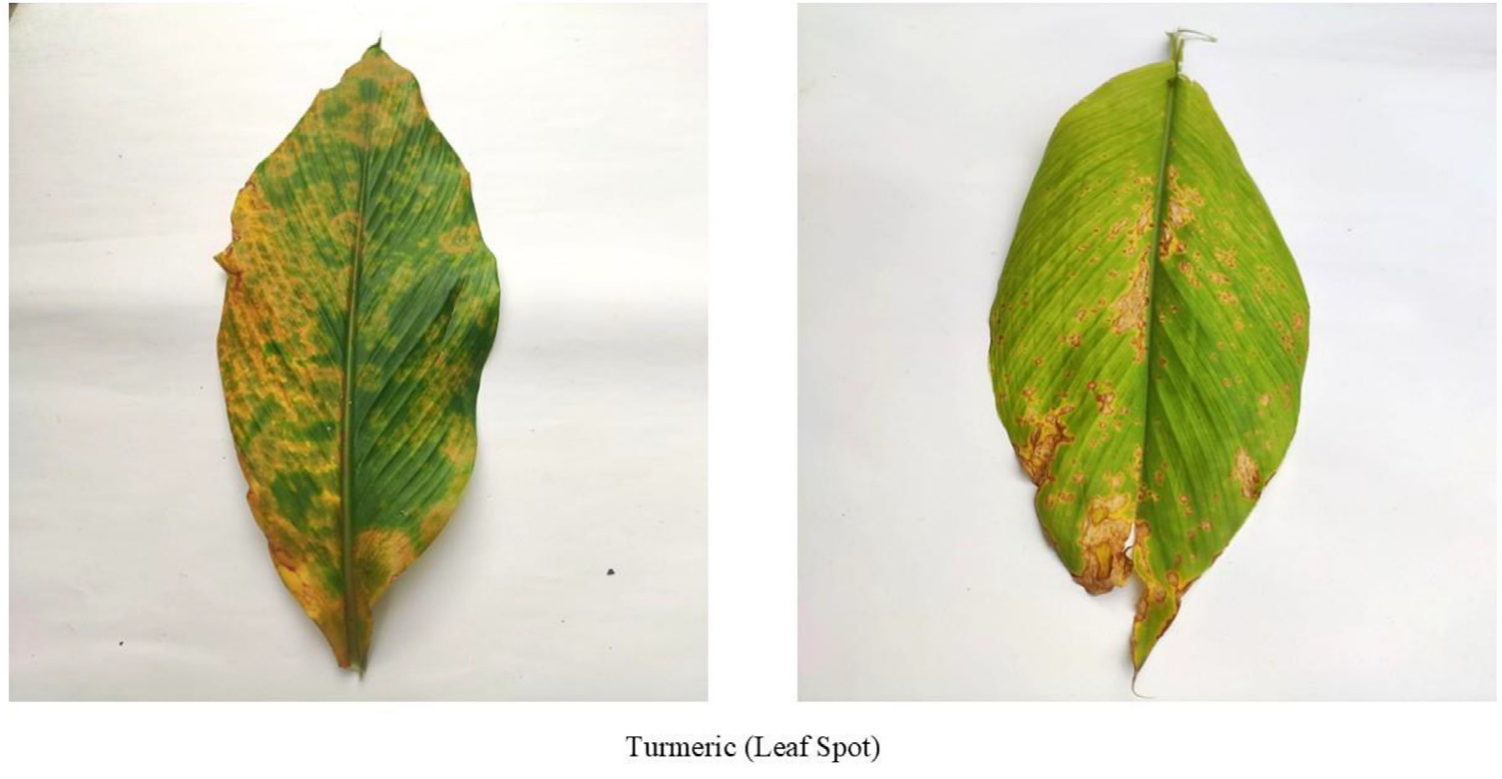
Turmeric leaf affected by Leaf Spot disease.

Fig. 4 illustrates turmeric leaf spot, a multiaxial phytopathological syndrome caused by various pathogens and environmental factors [13]. Colletotrichum spp. are cited as the primary causes of severe infections in areas [14]. The disease is more prevalent during the monsoon due to high humidity and the active vegetative phase of the plant, which reduces resistance and creates favorable microclimates. The first symptoms are irregular chlorotic to necrotic spots on the upper side of leaves. Lesion development depends on environmental conditions and pathogen species, ultimately reducing photosynthesis and crop yield.

**Fig. 5:**
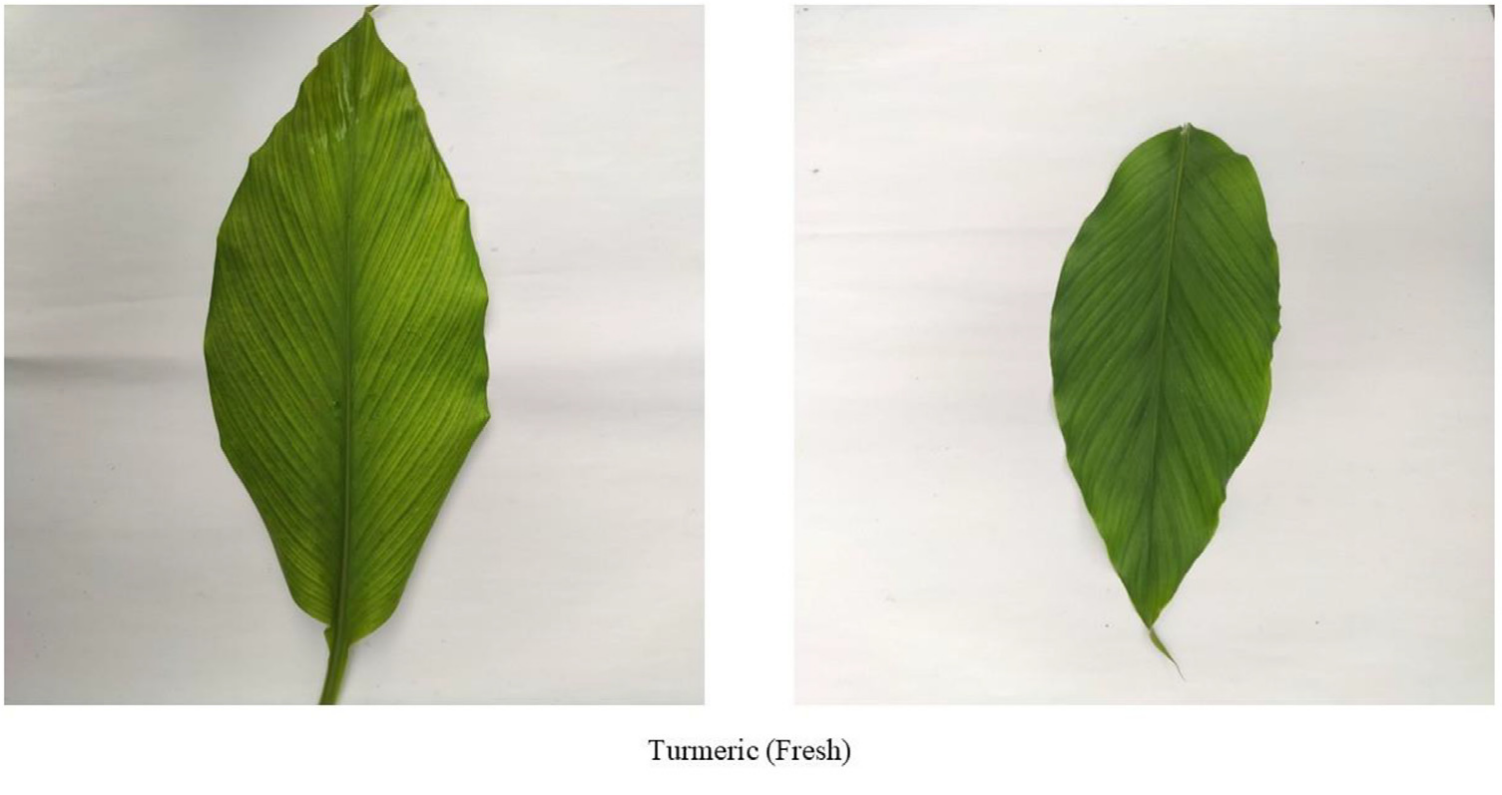
Turmeric healthy leaf.

Fig. 5 showcases healthy turmeric leaves, which display a deep, bright green color, smooth texture, and shiny surface. The leaf is elongated with a prominent central vein and clean edges, free of blemishes or discoloration. These healthy leaves are crucial for the plant’s growth, efficiently capturing sunlight for photosynthesis and producing solid rhizomes.

Table 1 offers an insightful comparison of several datasets that focus on the health conditions of turmeric leaves. The first dataset [15] features only one class for healthy and diseased leaves, with just 100 images. It lacks the necessary diversity to address the complexities of turmeric leaf diseases. Furthermore, the limited quantity of samples may lead to overfitting issues. Conversely, this dataset encompasses four classes: aphid disease, blotch, leaf spot, and healthy leaf, with a total of 865 images. This variety enhances the potential for robust model training. Such a dataset significantly contributes to deep learning research, particularly in developing reliable tools for early disease detection in agriculture.

**Table 1:** Dataset sample distribution.

Dataset	Number of Classes	Original Dataset	Augmented Dataset
Custodio, Epie [15]	1	100	–
This Dataset	4	865	3496

**Fig. 6:**
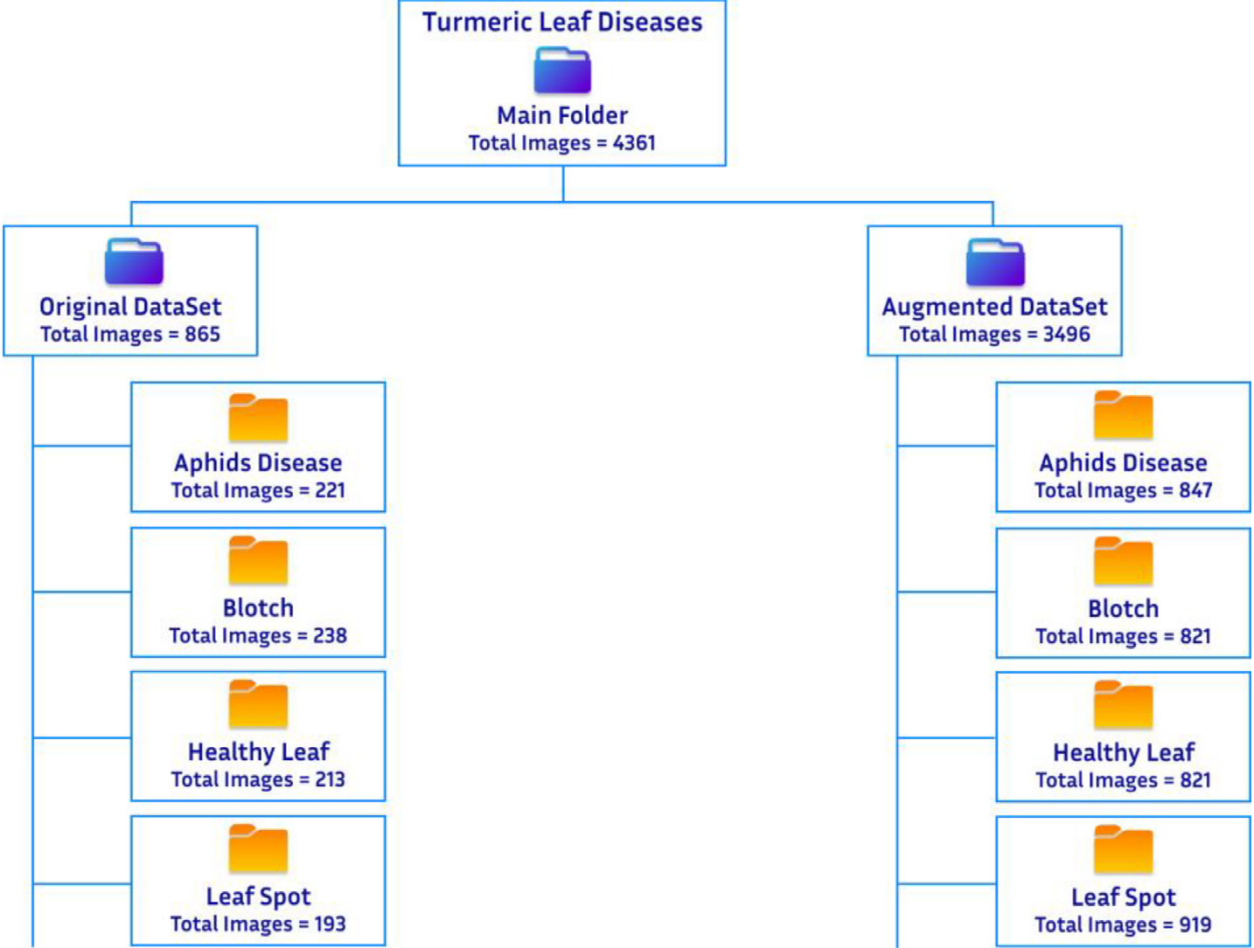
Overall dataset folder structure.

The turmeric leaf dataset is organized into two primary directories: the Original and Augmented datasets. Each directory is divided into four subfolders that represent specific conditions of turmeric leaves: Aphids Disease, Blotch, Leaf Spot, and Healthy Leaf. Table 2 illustrates the balanced distribution of images across each class, which boosts the dataset's suitability for model training and prevents the model from becoming biased toward any specific class. The original dataset comprises 865 images, while the augmented dataset contains 3496 images, all in JPG format. This hierarchical structure enables straightforward navigation and helps researchers efficiently select training and validation datasets, as demonstrated in Fig. 6. This organized dataset enables researchers to focus on refining algorithms for improved crop management and supports the exploration of advanced techniques in plant health analysis.

**Table 2:** Sample distribution and folder structure of the turmeric dataset.

Original Dataset			
Class	Category	No of images	Folder Name
1	Aphids Disease	221	Aphids Disease
2	Blotch	238	Blotch
3	Leaf Spot	193	Leaf Spot
4	Healthy Leaf	213	Healthy Leaf
	**Total**	865	
**Augmented Dataset**			
1	Aphids Disease	847	Aphids Disease
2	Blotch	909	Blotch
3	Leaf Spot	919	Leaf Spot
4	Healthy Leaf	821	Healthy Leaf
	**Total**	3496

## 4 Experimental Design, Materials, and Methods

**Fig. 7:**
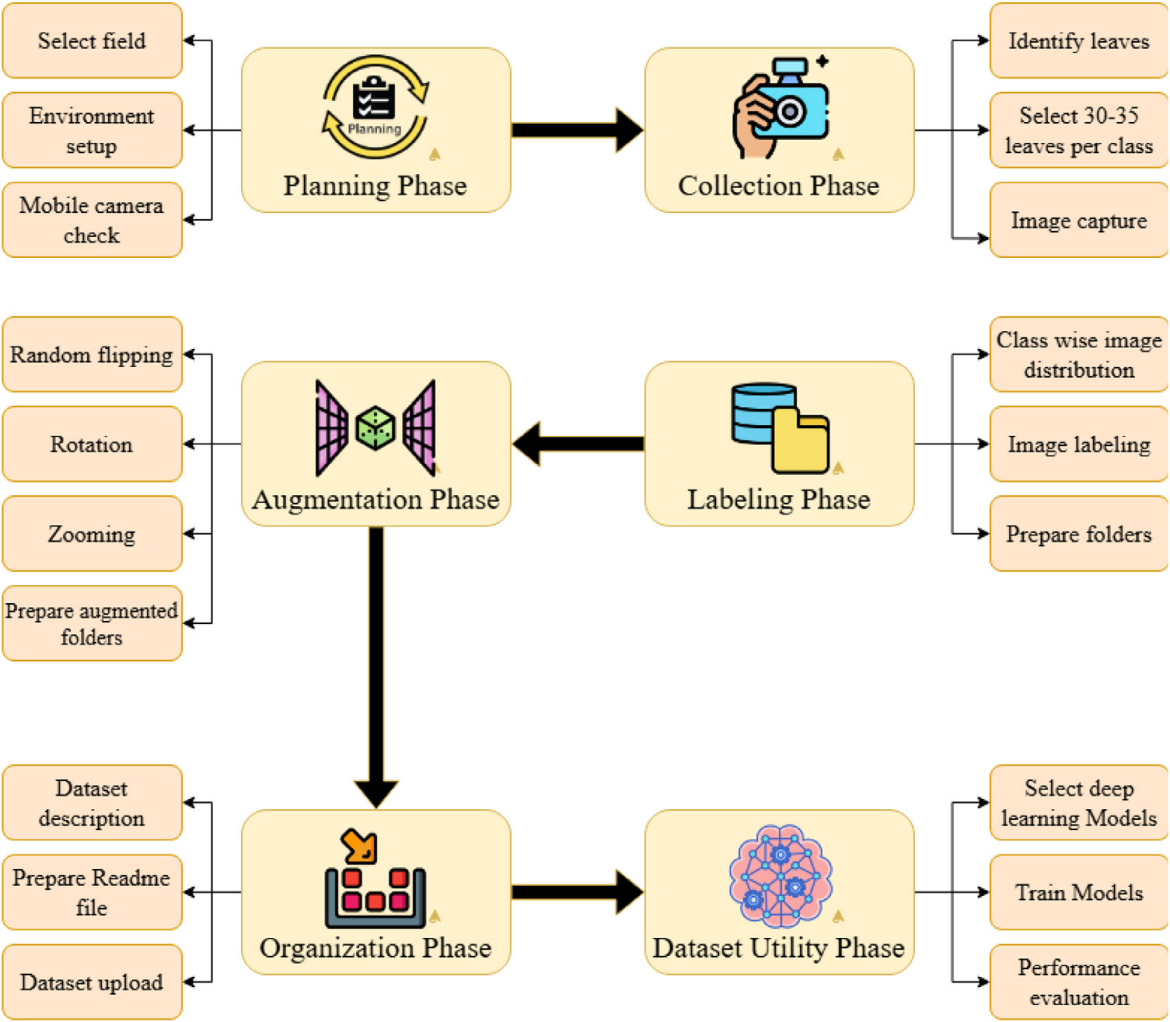
Overall workflow diagram for turmeric leaf.

The flowchart shown in Fig. 7 describes step-by-step turmeric leaf dataset collection and preprocessing methodology, separated into five main steps: Planning, Collection, Labeling and Structuring, Augmentation, and Organization. Under the Planning Phase, the methodology begins with the selection of an appropriate field where turmeric is grown. It is succeeded by environment preparation to maintain data collection consistency, and mobile device testing to maintain the quality and performance of the camera. Then, under the Collection Phase, turmeric leaves are individually picked in the field. Representative leaves, 30–35, are picked per category, and images are captured using a setup mobile arrangement, maintaining sample size balanced per category. The Label and Structure Phase is the separation of images per category to maintain structure-based labeling. Labeling is done correctly at this step to maintain correct classification when this model is to be trained and analyzed in the future. Under the Augmentation Phase, data augmentation in images is done using random flipping, rotation, and zooming with the purpose of artificially increasing dataset size and variety, hence the model, and maintaining improved robustness. When images are augmented, these are labeled once more in the correct manner to maintain consistency. Lastly, the Organization Phase is to organize the dataset. A dataset description is written in detail, and images are organized folder-wise to maintain easy reachability and usability. A README is also written to maintain the dataset creation process and metadata. The end-to-end procedure gives high-quality, well-structured, and augmented image data, which is now ready to be used in the application of machine learning in turmeric leaf classification or disease detection.

### 4.1 Dataset collection

Turmeric leaf disease dataset [7] was gathered systematically by making strenuous field visits across four diverse locations in the Pabna district, and four different varieties of diseases: aphid disease, blotch, leaf spot, and healthy leaves. The primary objective of the data collection process was to gather a diversified and representative dataset of images of turmeric leaves for easy identification and classification of diseases. The research methodology adopted a systematic process to ensure accuracy, consistency, and usability for deep learning applications.

Before the initiation of each field visit, turmeric plants were thoroughly examined to identify diseased and healthy leaves. Identification was aided through online sources and discussion with agricultural experts to ensure the presence of various diseases. This procedure was critical in picking a diverse variety of diseased leaves, which had more than one form of severity variation. Every selected leaf contained distinctive features that helped develop a strong dataset with diverse stages of diseases. For each class, we have selected approximately 30 to 35 distinct leaves from the turmeric plant based on the outward appearance of health, representing different patterns of disease. To maintain consistency and eliminate environmental background noise, the leaves were placed on a white background paper before image capture. The approach enhanced the clarity of the images and allowed the model to focus on the texture, structure, and disease symptoms of the leaf. Each leaf was imaged from five different angles, a critical step to increase dataset variability and improve the model's ability to generalize across varying conditions. This multi-view strategy captured variations in leaf orientation, illumination, and disease appearance, thus strengthening the dataset's resistance for deep learning use.

For clear picture acquisition, the Redmi K20 mobile phone was utilized for capturing the pictures, as depicted in Table 3. The phone's triple-camera and high-resolution features ensured proper and high-quality picture documentation. Nevertheless, challenges were encountered while collecting images, especially from natural light, which often caused overexposure and image saturation. To counter this problem, the levels of lighting were closely monitored and adjusted where required to reduce any negative effect on image quality. Since high-quality images are required for the effective detection of diseases, capturing them in an ideal environment was given importance. Various factors such as light, background preparation, and camera settings were taken into consideration to produce images with high resolution.

**Table 3:** Redmi K20 camera specification.

Camera Setup	Triple
Resolution	48 MP f/1.8, Primary Camera, 13 MP f/2.4, Ultra-Wide-Angle Camera, 8 MP f/2.4, Telephoto Camera
Sensor	IMX586, Exmor-RS CMOS Sensor
Autofocus	Phase Detection autofocus
Flash	Dual LED Flash
Settings	Exposure compensation, ISO control

To serve this purpose, a laboratory environment was specially designed with balanced light conditions to prevent too much light and shadow. A white background was employed to enhance natural surface features and prevent strong color contrast, rendering all images consistent. Furthermore, a high-resolution camera system was employed to capture images with accurate color gradients, preventing inconsistencies and producing an exhaustive data set. To eliminate environmental disturbances such as wind, leaves were collected and imaged under a controlled indoor setting to imbue the images with stability. After optimizing the conditions, multiple exposures were made from five different angles of every leaf, with comprehensive documentation and uniformity that is crucial to successful image analysis.

After the data collection process, the second important task was a rigorous selection process to determine the most appropriate images to use in machine learning. Only disease feature images that were clear and distinct were kept to enable accurate classification by machine learning models. The images were then well organized and categorized into specific folders after selection to enable an easy and systematic retrieval process. Additional purification was performed during the editing stage to promote clarity and consistency, rendering the dataset ideal for deep learning model training. This methodical and systematic data acquisition process guarantees that the turmeric leaf disease detection model is trained on high-quality and dependable information, thereby improving the accuracy and effectiveness of disease classification. A total of 865 images were gathered from four field trips with variations in angles, environmental conditions, and types of diseases. The dataset obtained is a valuable collection for training deep learning models in identifying and classifying turmeric leaf diseases accurately. Supplemented by a systematic pipeline, multi-variate samples, and standardized image capture, this dataset promises to play a very important role in plant disease detection research by employing state-of-the-art deep learning methodologies.

### 4.2 Dataset preparation

In an attempt to render the turmeric leaf disease diagnosis model more robust and general, several data augmentation techniques were performed on the dataset based on recent studies [16,17]. The augmentations are designed to avoid overfitting by adding various variations of the training set so that the model can generalize well under natural conditions. Geometric transformations such as random flipping, cropping, rotating, stretching, and zooming were particularly chosen to create realistic leaf orientation variation in the real world. These transformations are used to subject the model to a broad spectrum of leaf appearances without introducing unrealistic modifications that would otherwise weaken its performance. With such augmentation of the data, the model is made more adaptable to diverse orientations of leaves to be found in the field.

Along with the geometric transformations, color space changes were added to account for lighting conditions and color variations that are prevalent in natural conditions. Randomization of RGB channels, contrast, and brightness made the model more robust to different lighting and color conditions. This step guarantees the ability of the model to detect diseases under different environmental conditions. Additionally, the introduction of kernel filters, including sharpening and blurring operations, enhanced the model's capacity to handle images with varying degrees of image sharpness, resolving the problem of non-uniform image sharpness. These changes were especially successful in optimizing the model's disease detection capacity even when images are fuzzy or blurred, a prevalent problem in real-world applications.

Also contributing to the diversity of the dataset, random erasing was applied to simulate real-world conditions where parts of an image are occluded or missing. This practice compels the model to focus on the relevant features in the remaining parts of the image, thereby strengthening its ability to generalize to incomplete data. Apart from that, a mixing images technique was employed to combine multiple images to create novel composite samples, allowing the model to learn from complex variations and disease signs. These augmentations collectively ensured that the turmeric leaf disease detection model was trained on a varied, high-quality dataset, improving its ability to generalize well to different disease conditions and environmental factors.

Geometric transformations [17] are essential techniques used to enhance the robustness of a model by introducing variations in the orientation and appearance of objects, in this case, leaves. The following transformations were applied:

• **Random Flipping:** Horizontally or vertically flipping images to create mirrored versions, ensuring the model learns to recognize leaf structures regardless of orientation.
• **Rotation:** Rotating the image at different angles to mimic the natural movement of leaves in different environments, making the model adaptable to various perspectives.
• **Zooming:** Adjusting the scale of the image by zooming in or out to simulate different distances, allowing the model to analyze leaves at varied scales and maintain accuracy.

By implementing these transformations, the model becomes more resilient to variations in leaf orientation, improving its ability to recognize and classify leaves in diverse real-world settings. Let me know if you’d like further elaboration on any of these techniques!

### 4.3 Dataset use case

The turmeric leaf dataset was used to train and evaluate deep learning models for disease detection. This study implemented and trained two deep learning models, EfficientNetB7 and ResNet152, to classify the leaf conditions effectively. The dataset includes high-resolution images of healthy and diseased leaves of turmeric plants that are grown under natural conditions. The dataset enabled the models to learn image patterns about various conditions of leaves and to classify and analyze them precisely.

#### 4.3.1 EfficientNetB7 model

EfficientNetB7 is one of the highest variants in the EfficientNet series of convolutional neural networks, which is well-known for its superior performance and efficiency. The compound scaling approach is used to design the model, where depth, width, and resolution are scaled uniformly to achieve better performance. EfficientNetB7 utilizes depthwise separable convolutions and squeeze-and-excitation blocks to reduce computational cost without sacrificing accuracy. Pre-trained on huge datasets such as ImageNet, it is well-suited for transfer learning to other image classification applications. The model contains >66 million parameters, with a very high representational capacity. The model is designed for quick inference despite high complexity, on GPUs. EfficientNetB7 works extremely well in applications where very high accuracy is needed, such as medical imaging and plant disease identification. In this study, it was utilized for leaf disease classification because of its ability to capture fine-grained details. Bogireddy and Murari [18] suggested, in their 2024 study, the coupling of U-Net for segmentation of leaves and EfficientNetB7 for classification as a hybrid deep learning model, thus improving potato leaf disease detection. This joint method reached high accuracy and outperformed conventional CNN models, showing the strength of integrating segmentation and classification for accurate plant disease diagnosis.

#### 4.3.2 ResNet152 model

ResNet152 is a deep convolutional neural network architecture in the Residual Network (ResNet) family, which is well-known for its superior performance on image classification tasks. Proposed by He et al. (2016) [19], ResNet152 has 152 layers and introduces a new concept called residual learning. This method mitigates the degradation issue in deep networks by allowing layers to learn residual mappings instead of trying to learn the desired underlying functions directly. This is achieved using shortcut (or skip) connections that skip one or more layers, making it possible to train the network effectively even at extreme depths. The network features a stack of residual blocks, where each residual block involves convolutional layers followed by batch normalization and ReLU activation. ResNet152 is pre-trained on the ImageNet dataset, making it very effective for transfer learning applications. Its ability to extract deep hierarchical features makes it highly desirable for challenging image analysis tasks, such as plant disease detection. In this research, ResNet152 was used to classify leaf diseases, utilizing its depth and residual structure to identify complex patterns and textures in leaf images.

#### 4.3.3 Dataset split and hyperparameter

The training dataset of the EfficientNetB7 and ResNet152 models was split into 70 % for training, 20 % for validation, and 10 % for testing to provide effective learning, tuning, and unbiased performance measurement. Table 4 provides the major hyperparameters applied to both models. A dropout rate of 0.3 was used to avoid overfitting, while the optimizer's default learning rate was kept to ensure stable training. Categorical Cross-entropy was chosen to be the loss function, suitable for multi-class classification problems. A batch size of 32 allowed efficient processing of training data, and the models were trained for 25 epochs. To improve generalization and avoid overfitting, Early Stopping with a patience value of 5 was implemented as a regularization strategy.

**Table 4:** Hyperparameter for EfficientNetB7 and ResNet152.

Hyperparameter	Value
Dropout rate	0.3
Learning rate	Default
Loss Function	Categorical Crossentropy
Batch size	32
Epochs	25
Regularization	Early Stopping (patience = 5)

This consistent and thoughtful approach the tuning hyperparameters helped ensure that both models were trained under optimal and comparable conditions, contributing to reliable performance evaluation.

#### 4.3.4 Model evaluation

**Fig. 8:**
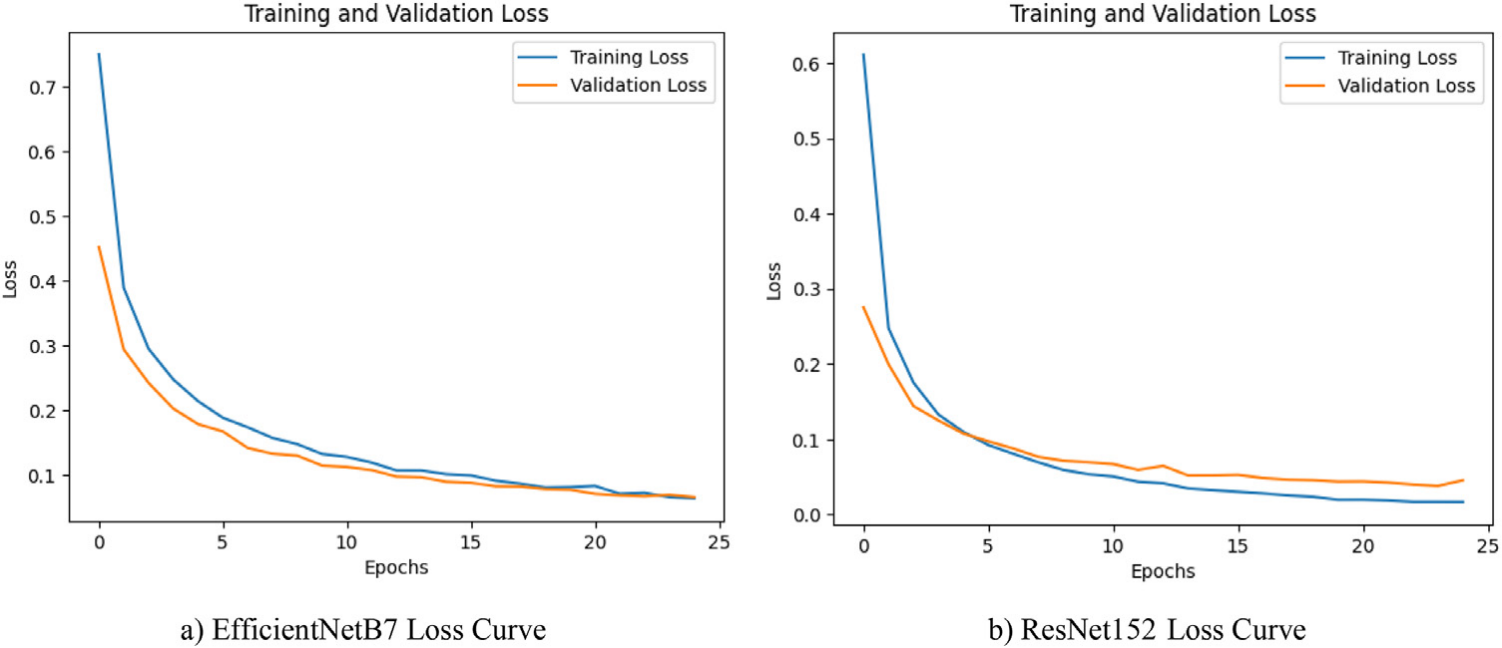
Models Loss Curves for Augmented Turmeric Dataset.

The loss curves indicated in Fig. 8 are the training and validation losses of the EfficientNetB7 and ResNet152 models when the models were trained on the turmeric dataset. From Fig. 8a, EfficientNetB7′s training loss and validation loss curves are seen to follow a consistent and stable drop throughout the epochs. Training loss starts at around 0.75 and validation loss at around 0.45. The losses fall precipitously within the first 5 epochs and plateau gradually, going as low as under 0.1 at epoch 25. The closeness between training loss and validation loss during training is seen as evidence of excellent generalization and little overfitting. EfficientNetB7 is extremely efficient in learning and is very resistant to tracking the turmeric leaf dataset. But, as can be seen from Fig. 8b, the ResNet152 plot, both the training loss and validation loss commence slightly lower than EfficientNetB7′s, at 0.6 and at around 0.3, respectively. The losses drop precipitously and reach stability around epoch 10. Both losses are at values very close to 0.02 at epoch 25, with very little difference between the two readings. It shows that ResNet152 is also very good at learning from the dataset and possesses excellent performance in generalization. Both networks are very good, with consistent declines in both training loss and validation loss, as well as little detection of overfitting. But ResNet152 can reach a lower terminal loss, which can indicate that it learned more fine-grained features from the dataset. EfficientNetB7, while being slightly behind as per terminal loss, still possesses very good performance and efficiency, which can be very desirable in conditions of scarcity of resources.

The performance metrics in Table 5 present the efficiencies of EfficientNetB7 and ResNet152 models on the turmeric leaf dataset.

**Table 5:** Model performance for turmeric augmented dataset.

Dataset	Accuracy ( %)	Precision ( %)	Recall ( %)	F1-Score ( %)
EfficienNetB7	98.67	98.60	98.66	98.62
ResNet152	97.87	98.12	97.97	98.04

EfficientNetB7 achieved a higher accuracy rate of 98.67 %, compared to the ResNet152 model's accuracy rate of 97.87 %. EfficientNetB7 recorded a precision rate of 98.60 %, slightly higher than ResNet152′s precision rate of 98.12 %, hence presenting a decrease in false positives. Similarly, EfficientNetB7 achieved a recall of 98.66 % as opposed to 97.97 % for ResNet152, indicating an improved detection of true positives. The F1-score, being a compromise between precision and recall, was 98.62 % for EfficientNetB7 and 98.04 % for ResNet152. As seen from the results, while the two models fared well, EfficientNetB7 performed slightly better in all the evaluation metrics, attesting to its applicability in the accurate classification of turmeric leaf diseases.

**Fig. 9:**
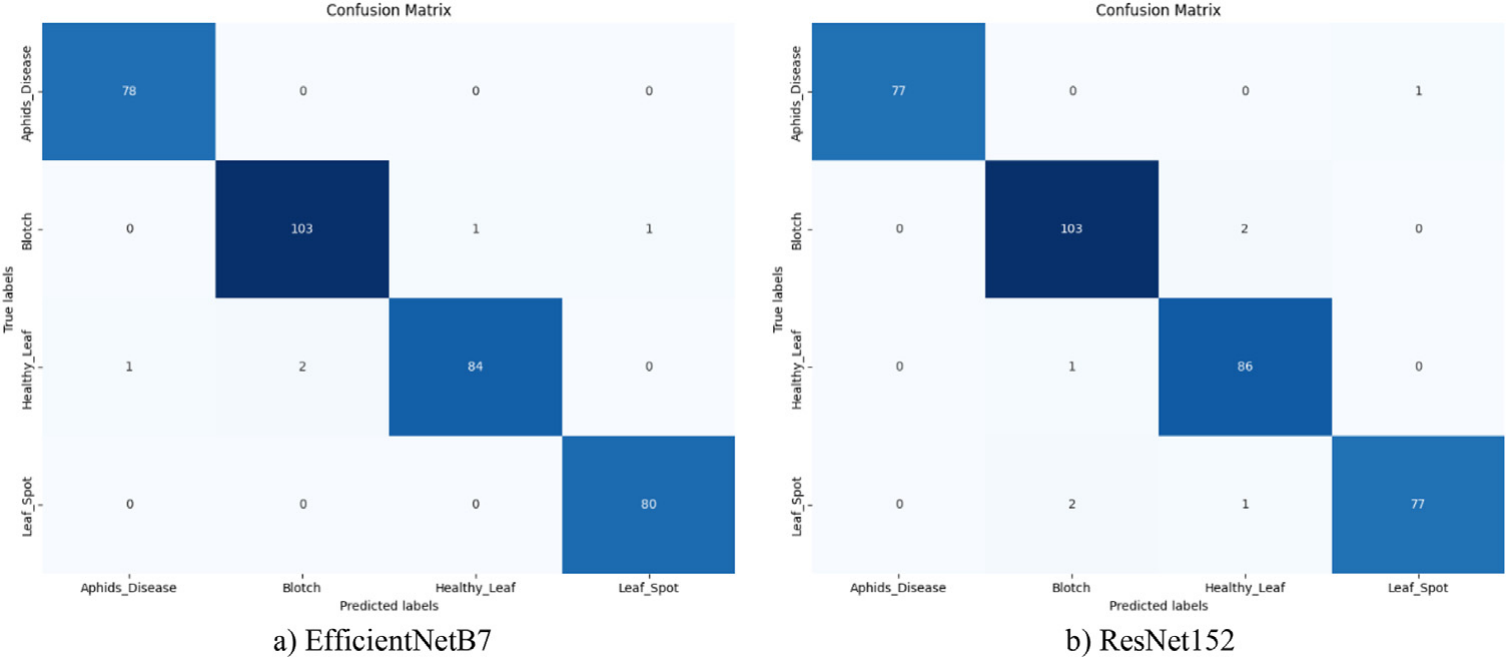
Models' confusion matrix for augmented turmeric dataset.

Fig. 9 illustrates the confusion matrices of EfficientNetB7 and ResNet152 models on the turmeric leaf dataset. The matrices indicate the classification performance on four classes: Aphids Disease, Blotch, Healthy Leaf, and Leaf Spot. EfficientNetB7 classified nearly all samples correctly with a few misclassifications, i.e., 2 samples of Healthy Leaf were misclassified as Blotch and 1 as Aphids Disease. On the other hand, the ResNet152 model was a bit more confused, especially misclassifying 2 Blotch samples and 1 Healthy Leaf as Leaf Spot. Both models had good class-wise predictions; however, EfficientNetB7 had fewer classification errors in general. Diagonal dominance in both matrices suggests good model accuracy, with EfficientNetB7 showing more clarity and class distinction. Table 6 provides further details of the confusion matrix.

**Table 6:** Models class wise prediction accuracy.

Model Name	Aphids Disease	Blotch	Leaf Spot	Healthy Leaf
EfficientNetB7	100 %	98 %	96.5 %	100 %
ResNet152	98.7 %	98 %	98.8 %	96.2 %

Table 6 presents the class-wise classification accuracy of the deep learning networks EfficientNetB7 and ResNet152 employed in turmeric leaf classification. EfficientNetB7 is observed to perform well by achieving 100 % accuracy in Aphids Disease and Healthy Leaf classification, which reflects the excellent ability of this network to differentiate between diseased leaves and healthy leaves. It also distinguishes between Leaf Spot and Blotch with very good accuracies of 98 % and 96.50 %, respectively. ResNet152, in contrast to this, is observed to be marginally better in Leaf Spot classification with 98.80 % accuracy, but as good as EfficientNetB7 in identifying Blotch (98 %). It is comparatively poorer in identifying Aphid Disease (98.70 %) and Healthy Leaf (96.20 %) than EfficientNetB7. Overall, the networks are very precise in identifying the conditions of turmeric leaves, but EfficientNetB7 is very well balanced and consistent across the board, and hence highly reliable in applications involving healthy and disease leaf discrimination with high accuracy.

#### 4.3.5 Real-time turmeric leaf classifier

We have developed a turmeric leaf disease detection system using Gradio to enable early detection in precision agriculture using an affordable and user-friendly app. By combining a trained EfficientNetB7 model with Gradio's simple interface, the system allows farmers to upload or take leaf images using their mobile phones and get instant predictions about the presence of disease. The early warning system helps minimize crop loss by enabling timely intervention, thus proving especially useful in resource-poor farming communities. The solution takes smart farming to the next level by combining accessibility, affordability, and AI-powered diagnostics.

**Fig. 10:**
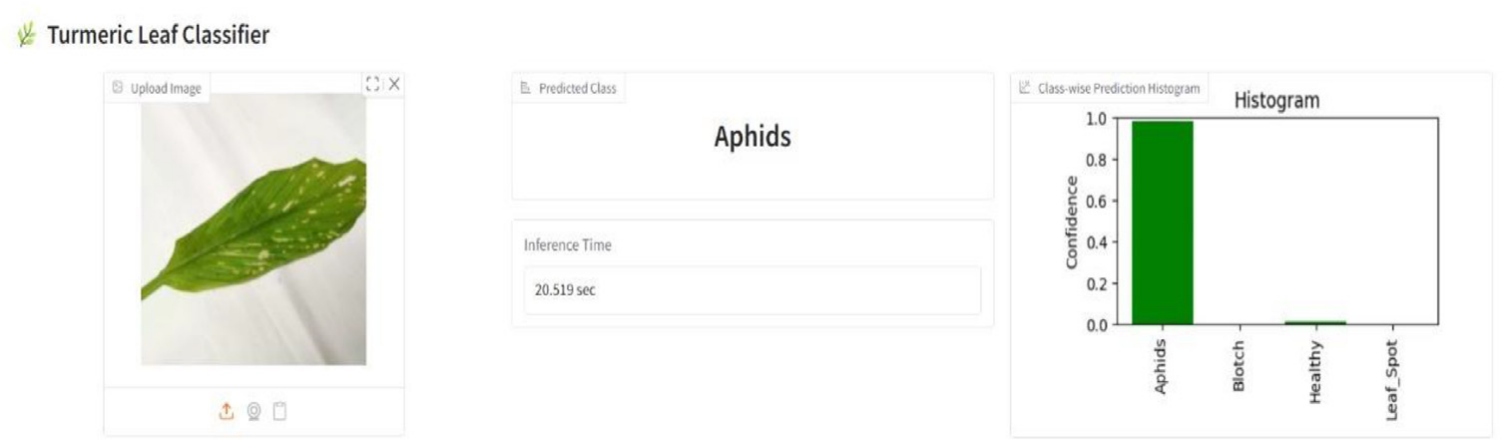
Turmeric leaf classifier.

Fig. 10 illustrates the user interface of the turmeric leaf disease classification system created using Gradio. The interface allows users to upload an image of a turmeric leaf, which a deep learning model for the type of disease present then analyzes. In this case, the predicted class is "Aphids," shown in the center of the interface along with the inference time of 20.519 s. On the right-hand side, a class-wise prediction histogram shows the confidence levels for all predicted classes Aphids, Blotch, Healthy, and Leaf Spot with the highest confidence (∼0.98) assigned to the Aphids class. This intuitive design enables quick and easy disease diagnosis for precision agriculture via an affordable and user-friendly AI solution.

## Limitations

During the collection of the turmeric leaf disease dataset, some issues affected both the diversity of the dataset and the reliability of the final model's accuracy. These limitations were addressed systematically to maintain the integrity of the dataset and ensure that the final model would be able to operate reliably in real-world scenarios. The major issues faced and how they were overcome are as follows:

• **Data Augmentation:** To make the model generalizable to various real-world conditions, data augmentation strategies were employed, including rotation, flipping, and brightness and contrast adjustment. This added variation to the dataset, making it easier for the model to identify leaves under varying environmental conditions. These strategies created a more stable dataset without requiring new data collection. However, additional augmentation techniques can be applied during training to further optimize performance and meet the requirements for deep learning models.
• **Environmental Variations and Image Quality**: The variations in image angles and lighting caused blurred or lopsided images, lowering detection accuracy. To avert this challenge, a controlled environment with constant lighting was provided, and correct angles for taking clear, high-quality images were utilized. This measure averted complications coming from uncontrolled environmental factors.
• **Diversification of Data:** The dataset needed to gather a plethora of turmeric leaves with varying disease symptoms and growing conditions. Gathering leaves of varying sizes, colors, and stages of the disease helped ensure that the model would be able to detect diseases in varying situations. This diversification helped the model generalize well to a plethora of real-life cases.
• **Ethical and Legal Considerations**: Ethical issues were dealt with by seeking permission from farmers before leaf collection. This helped in upholding good rapport with the farming community as well as legal compliance. Ethical standards ensured responsible and respectful data collection.
• **Seasonal and Geographic Constraints**: The prevalence of diseases was seasonal and geographical, which affected the coverage of some diseases in the dataset. To overcome this, data from various regions were collected to have a representative dataset for a variety of disease conditions. This enabled the model to generalize well across environments. This study focuses on identifying and visually collecting turmeric leaf disease images within a specific environmental setting. While this approach ensures consistency in data acquisition, it limits the dataset's diversity across different environmental conditions.
• **Limited Data Availability:** There was no large turmeric leaf disease dataset available beforehand, and thus, the images had to be collected and annotated manually, which was a time-consuming process. Data augmentation techniques were applied to make up for the low data, which increased the dataset without further manual collection. This enabled the model to train on a larger variety of samples.
• **Image Annotation and Labeling:** Annotation of images with the right disease labels was tricky owing to the complexity and subtlety of differences in symptoms. A rigorous, expert-led annotation process was followed to avoid inaccuracies and errors. Proper labeling was extremely important to construct an effective model, as misannotations would cause degraded training performance.

## Ethics Statement

The authors adhere to the journal's ethical guidelines and confirm that this research does not involve humans, animals, or data obtained from social media. The datasets utilized in the study are publicly accessible, and appropriate citation protocols should be followed when utilizing these datasets.

## Credit Author Statement

**Jubaer Ahmed:** Conceptualization, Methodology, Supervision, Visualization, Project administration, Validation, Writing – review & editing; **Md. Riyad Hossain:** Investigation, Data collection, Methodology, Writing – original draft, Writing – review & editing; **Raiyan Gani:** Conceptualization, Methodology, Supervision, Visualization, Project administration, Validation, Writing – review & editing; **Mohammad Rifat Ahmmad Rashid:** Conceptualization, Methodology, Supervision, Visualization, Project administration, Validation, Writing – review & editing; **Md. Mahamudur Rahman:** Investigation, Data collection, Methodology, Writing – original draft, Writing – review & editing; **Tasfia Binte Jahangir:** Investigation, Data collection, Writing – original draft, Writing – review & editing; **Md. Samir Hossain:** Investigation, Data collection, Methodology, Writing – original draft, Writing – review & editing.

## Declaration of Competing Interest

The authors declare that they have no known competing financial interests or personal relationships that could have appeared to influence the work reported in this paper.

## Data Availability

Mendeley DataImage Dataset for Turmeric Plant Leaf Disease Detection (Original data)
